# Measurement of Net NH_4_^+^ Fluxes Using the Non-invasive Micro-Test Technology (NMT) System in Rice

**DOI:** 10.21769/BioProtoc.5773

**Published:** 2026-08-05

**Authors:** Dong-Wei Di, Yunqi Liu, Bin Ye, Weiming Shi

**Affiliations:** 1 State Key Laboratory of Soil and Sustainable Agriculture, Institute of Soil Science, Chinese Academy of Sciences, Nanjing, China; 2 University of Chinese Academy of Sciences, Beijing, China; 3 Zhongguancun Xuyue Non-invasive Micro-test Technology Industrial Alliance, Beijing, China; 4 Xuyue (Beijing) Sci. & Tech. Co., Ltd., Beijing, China

**Keywords:** NH_4_^+^ fluxes, NMT, Root, Protoplast, Vacuole

## Abstract

Ammonium (NH_4_^+^) is the primary inorganic nitrogen source for rice (*Oryza sativa* L.). Substantial progress has been made in characterizing the functions of ammonium transporters (AMTs) in roots; however, the regulatory dynamics governing subcellular ammonium compartmentation after its entry into cells, particularly its vacuolar sequestration and efflux back to the external environment, remain poorly understood. This knowledge gap stems mainly from two factors: the difficulty of applying conventional detection methods at the organellar scale and interference caused by nonspecific ion adsorption to the cell wall of intact roots. To address these challenges, we present a detailed and reproducible protocol for real-time measurement of net NH_4_^+^ fluxes in rice roots, root protoplasts, and isolated vacuoles using non-invasive micro-test technology (NMT). The protocol covers the preparation of protoplasts and vacuoles from rice roots, the configuration and calibration of the NMT system, and the step-by-step measurement of net NH_4_^+^ fluxes at three distinct biological levels (intact roots, protoplasts, and vacuoles). By employing a unified sample preparation and measurement strategy, this protocol enables quantification of net uptake fluxes across the plasma membrane, characterization of net efflux dynamics under specific conditions, and indirect estimation of vacuolar sequestration capacity using the isolated vacuole system. Overall, this protocol provides a flexible and robust framework for studying NH_4_^+^ homeostasis in plants and is readily adaptable to different crop species, treatment conditions, and experimental objectives. Owing to its modular design and compatibility with standard NMT equipment, it can be readily adopted by laboratories seeking to investigate nitrogen transport mechanisms in plants.

Key features

• Allows for testing of NH_4_^+^ fluxes in roots, protoplasts, and vacuoles.

• Applicable to plants grown under different culture systems, including *Arabidopsis thaliana* grown in dishes and rice grown in hydroponic systems.

• Supports both long-term and transient stress treatments.

• Real-time monitoring.

## Graphical overview


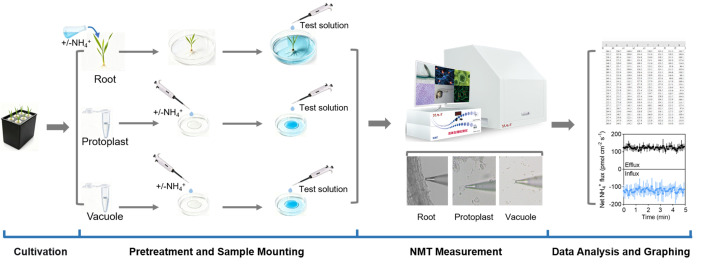


## Background

Ammonium (NH_4_^+^) is the primary inorganic nitrogen source for rice (*Oryza sativa* L.) [1]. The root system acts as the main gateway for NH_4_^+^ entry, where plasma membrane–localized ammonium transporters (AMTs) facilitate its efficient uptake into root cells [2,3]. Much of what we know about net NH_4_^+^ uptake at the whole-root level and AMT function comes from physiological and molecular genetic studies. By contrast, the regulatory mechanisms that control subcellular NH_4_^+^ compartmentation, especially vacuolar storage and efflux back out of the cell, are still largely obscure [4].

The vacuole is the largest organelle in mature rice root cells, often occupying >80% of the total cell volume [5]. Vacuolar NH_4_^+^ storage not only buffers cytoplasmic free NH_4_^+^ to prevent toxicity but also contributes critically to nitrogen storage, remobilization, and stress adaptation [6,7]. However, owing to the limitations of conventional methods at the organellar scale, quantifying the dynamic partitioning of NH_4_^+^ between the cytoplasm and the vacuole has posed a persistent technical challenge. Consequently, direct evidence is still lacking for how vacuolar compartmentation coordinates with cytoplasmic NH_4_^+^ detoxification and plasma membrane efflux.

Futile NH_4_^+^ efflux is a key electrophysiological mechanism underlying NH_4_^+^ toxicity in plants [8–10]. Existing evidence indicates that the magnitude of NH_4_^+^ efflux is negatively correlated with NH_4_^+^ tolerance, uptake efficiency, and nitrogen use efficiency in rice, suggesting that restricting efflux may be an effective strategy to concurrently improve these traits [10–12]. Thus, elucidating the regulatory mechanisms of NH_4_^+^ efflux is of major theoretical importance, which depends heavily on accurate quantification of transmembrane net NH_4_^+^ fluxes. Dissecting how regulatory proteins influence net efflux phenotypes requires a technique capable of real-time net flux measurement. A major obstacle, however, is the nonspecific adsorption of ions by the cell wall of intact roots, which severely compromises measurement accuracy. Therefore, in addition to measuring net NH_4_^+^ fluxes in intact roots, a system that eliminates cell wall interference is needed, specifically the direct measurement of net NH_4_^+^ fluxes around protoplasts. Of note, non-invasive micro-test technology (NMT) measures net ion fluxes, which primarily reflect net efflux dynamics [13]. For precise resolution of unidirectional fluxes, complementary approaches such as isotopic tracing or patch-clamp electrophysiology are required [14].

To address these technical bottlenecks, we developed an integrated analytical pipeline combining rice roots, root protoplasts, and isolated vacuoles. Protoplasts, plant cells that retain intact plasma membrane and tonoplast after cell wall removal, eliminate the physical barrier of the cell wall to solute exchange and enable precise control over the ionic composition and osmotic pressure of the external solution. By comparing NH_4_^+^ transport characteristics across the three levels (intact roots, protoplasts, isolated vacuoles), we can quantify net uptake across the plasma membrane, assess net efflux dynamics under defined conditions (e.g., transient high external NH_4_^+^), and indirectly evaluate vacuolar compartmentation capacity using the isolated vacuole system. Here, we provide detailed procedures for preparing protoplasts and vacuoles from rice roots, along with a method for real-time net NH_4_^+^ flux measurement based on NMT. By preserving the integrity and activity of each membrane system, this protocol allows independent dissection of net NH_4_^+^ transport events at distinct cellular levels, offering a robust methodological platform for elucidating the NH_4_^+^ homeostasis network in rice and laying a technical foundation for improving nitrogen use efficiency and NH_4_^+^ tolerance in crops.

## Materials and reagents

**Biological materials**

1. Rice seedlings

**Reagents**

1. Yoshida rice nutrient salts (Coolaber, catalog number: NSP1040)

2. Tris(hydroxymethyl)aminomethane (Tris) (Sinopharm Chemical Reagent, catalog number: 69097-20-7)

3. Hydrochloric acid (HCl) (Sinopharm Chemical Reagent, catalog number: 7647-01-0)

4. 2-(N-morpholino)ethanesulfonic acid (MES) (BioFroxx, catalog number: 145224-94-8)

5. 4-(2-hydroxyethyl)-1-piperazineethanesulfonic acid (HEPES) (Yuanye, catalog number: 7365-45-9)

6. Sodium hydroxide (NaOH) (Sinopharm Chemical Reagent, catalog number: 1310-73-2)

7. Ammonium chloride (NH_4_Cl) (Sinopharm Chemical Reagent, catalog number: 12125-02-9)

8. Ammonium nitrate (NH_4_NO_3_) (Sinopharm Chemical Reagent, catalog number: 6484-52-2)

9. Calcium chloride (CaCl_2_) (Sinopharm Chemical Reagent, catalog number: 10043-52-4)

10. Mannitol (Sinopharm Chemical Reagent, catalog number: 69-65-8)

11. Dipotassium EDTA (K_2_EDTA·2H_2_O) (Sinopharm Chemical Reagent, catalog number: 2001-94-7)

12. Protoplast Isolation Kit [Real-Times (Beijing) Biotechnology Co., Ltd., catalog number: RTU4082]

13. Hydrogen peroxide 30% (H_2_O_2_) (Sinopharm Chemical Reagent, catalog number: 7722-84-1)

**Solutions**

1. Rice culture solution (see Recipes)

2. Protoplast storage solution (see Recipes)

3. Vacuolar storage solution (see Recipes)

4. Test solution for NH_4_^+^ influx (see Recipes)

5. Calibration solution for NH_4_^+^ influx (see Recipes)

6. Test solution for NH_4_^+^ efflux (see Recipes)

7. Calibration solution for NH_4_^+^ efflux (see Recipes)

8. NH_4_^+^-free treatment solution (see Recipes)

9. High NH_4_^+^ treatment solution (see Recipes)

10. Tris solution (see Recipes)

11. HCl solution (see Recipes)

12. NaOH solution (see Recipes)

13. Hypotonic lysis buffer (see Recipes)

**Recipes**

**1. Rice culture solution**

**Table:** 

Reagent	Final concentration	Quantity or volume
Yoshida rice nutrient salts	n/a	0.535 g
Double-distilled water (ddH_2_O)	n/a	To 1 L
Total	n/a	1 L

Adjust pH to 5.5 using NaOH and HCl.

**2. Protoplast storage solution**

**Table:** 

Reagent	Final concentration	Quantity or volume
Mannitol	500 mM	9.1085 g
MES	2 mM	0.4265 g
ddH_2_O	n/a	To 100 mL
Total	n/a	100 mL

Adjust pH to 6.0 using Tris and HCl.

**3. Vacuolar storage solution**

**Table:** 

Reagent	Final concentration	Quantity or volume
Mannitol	500 mM	9.1085 g
HEPES	10 mM	0.2383 g
ddH_2_O	n/a	To 100 mL
Total	n/a	100 mL

Adjust pH to 7.0 using Tris and HCl.

**4. Test solution for NH_4_^+^ influx**

a. For roots

**Table:** 

Reagent	Final concentration	Quantity or volume
NH_4_NO_3_	1.5 mM	1.5 mL of 100 mM stock
CaCl_2_	0.1 mM	0.1 mL of 100 mM stock
MES	0.2 mM	0.2 mL of 100 mM stock
ddH_2_O	n/a	To 100 mL
Total	n/a	100 mL

Adjust pH to 5.8 using Tris and HCl. The solution should be prepared immediately before use.

b. For protoplasts

**Table:** 

Reagent	Final concentration	Quantity or volume
NH_4_Cl	1.5 mM	1.5 mL of 100 mM stock
CaCl_2_	0.1 mM	0.1 mL of 100 mM stock
Mannitol	500 mM	9.1085 g
MES	0.2 mM	0.2 mL of 100 mM stock
ddH_2_O	n/a	To 100 mL
Total	n/a	100 mL

Adjust pH to 6.0 using Tris and HCl. The solution should be prepared immediately before use.

c. For vacuoles

**Table:** 

Reagent	Final concentration	Quantity or volume
NH_4_Cl	4.0 mM	4 mL of 100 mM stock
Mannitol	500 mM	9.1085 g
HEPES	0.2 mM	0.2 mL of 100 mM stock
ddH_2_O	n/a	To 100 mL
Total	n/a	100 mL

Adjust pH to 7.0 using Tris and HCl. The solution should be prepared immediately before use.

**5. Calibration solution for NH_4_^+^ influx**

a. For roots

**Table:** 

Reagent	Final concentration	Quantity or volume
NH_4_NO_3_	0.15 mM	0.15 mL of 100 mM stock
CaCl_2_	0.1 mM	0.1 mL of 100 mM stock
MES	0.2 mM	0.2 mL of 100 mM stock
ddH_2_O	n/a	To 100 mL
Total	n/a	100 mL

Adjust pH to 5.8 using Tris and HCl. The solution should be prepared immediately before use.

b. For protoplasts

**Table:** 

Reagent	Final concentration	Quantity or volume
NH_4_NO_3_	0.15 mM	0.15 mL of 100 mM stock
CaCl_2_	0.1 mM	0.1 mL of 100 mM stock
Mannitol	500 mM	9.1085 g
MES	0.2 mM	0.2 mL of 100 mM stock
ddH_2_O	n/a	To 100 mL
Total	n/a	100 mL

Adjust pH to 5.8 using Tris and HCl. The solution should be prepared immediately before use.

c. For vacuoles

**Table:** 

Reagent	Final concentration	Quantity or volume
NH_4_Cl	0.4 mM	0.4 mL of 100 mM stock
Mannitol	500 mM	9.1085 g
HEPES	0.2 mM	0.2 mL of 100 mM stock
ddH_2_O	n/a	To 100 mL
Total	n/a	100 mL

Adjust pH to 7.0 using Tris and HCl. The solution should be prepared immediately before use.

**6. Test solution for NH_4_^+^ efflux**

a. For roots

**Table:** 

Reagent	Final concentration	Quantity or volume
NH_4_Cl	0.2 mM	0.2 mL of 100 mM stock
CaCl_2_	0.1 mM	0.1 mL of 100 mM stock
MES	0.2 mM	0.2 mL of 100 mM stock
ddH_2_O	n/a	To 100 mL
Total	n/a	100 mL

Adjust pH to 5.8 using Tris and HCl. The solution should be prepared immediately before use.

b. For protoplasts

**Table:** 

Reagent	Final concentration	Quantity or volume
NH_4_Cl	0.2 mM	0.2 mL of 100 mM stock
CaCl_2_	0.1 mM	0.1 mL of 100 mM stock
Mannitol	500 mM	9.1085 g
MES	0.2 mM	0.2 mL of 100 mM stock
ddH_2_O	n/a	To 100 mL
Total	n/a	100 mL

Adjust pH to 6.0 using Tris and HCl. The solution should be prepared immediately before use.

c. For vacuoles

**Table:** 

Reagent	Final concentration	Quantity or volume
NH_4_Cl	0.2 mM	0.2 mL of 100 mM stock
Mannitol	500 mM	9.1085 g
HEPES	0.2 mM	0.2 mL of 100 mM stock
ddH_2_O	n/a	To 100 mL
Total	n/a	100 mL

Adjust pH to 7.0 using Tris and HCl. The solution should be prepared immediately before use.

**7. Calibration solution for NH_4_^+^ efflux**

a. For roots

**Table:** 

Reagent	Final concentration	Quantity or volume
NH_4_Cl	2.0 mM	2.0 mL of 100 mM stock
CaCl_2_	0.1 mM	0.1 mL of 100 mM stock
MES	0.2 mM	0.2 mL of 100 mM stock
ddH_2_O	n/a	To 100 mL
Total	n/a	100 mL

Adjust pH to 5.8 using Tris and HCl. The solution should be prepared immediately before use.

b. For protoplasts

**Table:** 

Reagent	Final concentration	Quantity or volume
NH_4_Cl	2.0 mM	2.0 mL of 100 mM stock
CaCl_2_	0.1 mM	0.1 mL of 100 mM stock
Mannitol	500 mM	9.1085 g
MES	0.2 mM	0.2 mL of 100 mM stock
ddH_2_O	n/a	To 100 mL
Total	n/a	100 mL

Adjust pH to 6.0 using Tris and HCl. The solution should be prepared immediately before use.

c. For vacuoles

**Table:** 

Reagent	Final concentration	Quantity or volume
NH_4_Cl	2.0 mM	2.0 mL of 100 mM stock
Mannitol	500 mM	9.1085 g
HEPES	0.2 mM	0.2 mL of 100 mM stock
ddH_2_O	n/a	To 100 mL
Total	n/a	100 mL

Adjust pH to 7.0 using Tris and HCl. The solution should be prepared immediately before use.

**8. NH_4_^+^-free treatment solution**

**Table:** 

Reagent	Final concentration	Quantity or volume
CaCl_2_	0.1 mM	0.1 mL of 100 mM stock
Mannitol	500 mM	9.1085 g
MES	0.2 mM	0.2 mL of 100 mM stock
ddH_2_O	n/a	To 100 mL
Total	n/a	100 mL

Adjust pH to 6.0 using Tris and HCl. The solution should be prepared immediately before use.

**9. High NH_4_^+^ treatment solution**

**Table:** 

Reagent	Final concentration	Quantity or volume
NH_4_Cl	10 mM	10 mL of 100 mM stock
CaCl_2_	0.1 mM	0.1 mL of 100 mM stock
Mannitol	500 mM	9.1085 g
MES	0.2 mM	0.2 mL of 100 mM stock
ddH_2_O	n/a	To 100 mL
Total	n/a	100 mL

**10. Tris solution**

**Table:** 

Reagent	Final concentration	Quantity or volume
Tris	1 M	12.11 g
ddH_2_O	n/a	To 100 mL
Total	n/a	100 mL
Total	n/a	100 mL

**11. HCl solution**

**Table:** 

Reagent	Final concentration	Quantity or volume
HCl	1 M	8.3 mL
ddH_2_O	n/a	To 100 mL
Total	n/a	100 mL

**12. NaOH solution**

**Table:** 

Reagent	Final concentration	Quantity or volume
NaOH	1 M	4 g
ddH_2_O	n/a	To 100 mL
Total	n/a	100 mL

**13. Hypotonic lysis buffer**

**Table:** 

Reagent	Final concentration	Quantity or volume
HEPES	5 mM	0.119 g
Mannitol	100 mM	1.822 g
K_2_EDTA·2H_2_O	1 mM	0.0405 g
ddH_2_O	n/a	To 100 mL
Total	n/a	100 mL

Adjust pH to 7.0 using Tris and KOH. The solution should be prepared immediately before use.

**Laboratory supplies**

1. Petri dish (Biosharp, catalog number: BS-100-SD)

2. Culture pot (Taobao, Zesheng)

3. 50 mL centrifuge tube (Axygen, catalog number: SCT-50ML-25-S)

4. 1.5 mL centrifuge tube (Axygen, catalog number: MCT-150-C-S)

5. Cell strainer (Loikaw, catalog number: S-016802)

6. Cell culture coverslip (BKMAM, catalog number: 130213002)

7. 10–1,000 μL pipette tips (Kirgen, catalog numbers: KG1011, KG1212, KG1313)

8. Forceps

9. Blade

10. Purified water

11. Ice

12. Resin block (Taobao, catalog number: n/a)

## Equipment

1. Non-invasive micro-test system (Xuyue, model number: NMT Physiolyzer^®^)

2. NH_4_^+^ flux microsensor (Xuyue, model number: XY-STZ-NH4-C)

3. Centrifuge (Eppendorf, model number: 5804R)

4. Shaker (SHIPING, model number: DJS-2020)

5. Weighing balance with 0.0001 g accuracy (Metter Toledo, model: XS204)

6. Vacuum pump [YUHUA, model number: SHZ-D()]

7. Water bath (JINGHONG, model number: XMTD-8222)

8. pH meter (METTER TOLEDO, model number: 30997001)

## Software and datasets

1. imFluxes V3.0 software (Xuyue)

## Procedure

**A. Rice seed germination and cultivation**

1. Surface-disinfect rice seeds with 3% H_2_O_2_ for 30 min and then wash thoroughly with distilled water three times.

2. Place the seeds in the dark at 30 °C for 48 h to germinate.

3. After germination, evenly transfer the seeds onto floating nylon mesh nets and kept in a growth chamber.

4. For nutrient acclimation, first grow seedlings in 1/4-strength modified Yoshida solution for 2 days, followed by 1/2-strength solution for another 4 days. Finally, transfer to a full-strength solution for 6 days.

**B. Protoplast isolation**

1. Enzymatic digestion: Cut roots from 12-day-old rice seedlings in good growth condition into 1 mm segments. For every 20 seedlings (approximately 0.1 g), immediately transfer the segments to a 50 mL centrifuge tube containing 10 mL of enzyme digestion solution (Table 1) and fully immerse them. Evacuate the tube under vacuum for 30 min using a vacuum pump in the dark. Then, allow the tissue to digest in the dark at 25°C for 4 h, with gentle mixing at intervals.

*Note: Protoplast isolation from rice roots is notoriously challenging. For a single sample, roots from 30–40 seedlings are recommended, which should be cut into small pieces and submerged in 20 mL of enzyme solution. Vacuum infiltration helps the enzyme solution penetrate the intercellular spaces; however, this step may be skipped if a vacuum pump is not available.*

**Table 1.** Enzyme digestion solution. (Components from the Protoplast Isolation kit.)

Reagent	10 mL system	20 mL system
Enzyme dissolution buffer (2×)	5 mL	10 mL
Enzyme premix	0.019 g	0.038 g
Incubate in a 55°C water bath for 10 min with intermittent mixing. After cooling, add the following reagents:		
Reducing agent	5 μL	10 μL
50 mg/mL BSA	0.2 mL	0.4 mL
Sterile water	To 10 mL	To 20 mL

*Note: Prepare this solution fresh immediately before use; do not store it frozen.*

2. Rinsing: Following enzymatic digestion, gently mix an equal volume of 1× rinsing solution (Table 2) with the enzyme digestion solution.

**Table 2.** 1× rinsing solution. (Components from the Protoplast Isolation kit.)

Reagent	Volume for 10 mL
Rinsing solution (5×)	2 mL
Sterile water	8 mL

3. Filtration: Filter the mixture obtained in step B2 through a 70 μm cell strainer to remove undigested root tissues. Rinse the enzyme digestion centrifuge tube and the retained undigested roots one to two times with 10–20 mL of 1× rinsing solution (Table 2). Collect all filtrate and rinsing liquids into a 50 mL centrifuge tube.

4. First collection: Centrifuge the filtrate at 100× *g* for 2 min at room temperature and remove the supernatant as thoroughly as possible.

*Note: During centrifugation, reduce both the acceleration and deceleration rates. A high acceleration rate may cause protoplasts to adhere to the tube wall, while a high deceleration rate may resuspend the pelleted protoplasts from the bottom of the tube. Set both acceleration and deceleration to 3.*

5. Second collection: Add 5 mL of 1× rinsing solution (Table 2) to the pellet and resuspend the protoplasts using a 1,000 μL pipette tip. Then, incubate the suspension on ice for 30 min to allow protoplasts to settle to the bottom of the centrifuge tube by gravity. Remove the supernatant as much as possible and collect the protoplasts.

*Note: Alternatively, collect protoplasts by centrifugation at 100× g for 1–2 min at room temperature.*

6. Resuspension: Carefully remove the supernatant without disturbing the protoplast pellet. Then, resuspend the pellet in protoplast storage solution (Recipe 2) to obtain the protoplast suspension.

*Notes:*

*1. Protoplasts can be preserved for at least 24 h under ice-cold conditions.*

*2. Under the microscope integrated with the NMT system, protoplasts appear as intact, organelle-containing cells, whereas vacuoles are observed as empty, membrane-bound vesicles without visible internal structures.*

**C. Vacuole isolation**

*Note: Perform steps B1–5 exactly as described above.*

1. Hypotonic lysis and differential centrifugation purification: Gently resuspend the protoplast pellet collected in step B5 in 10 mL of ice-cold hypotonic lysis buffer (Recipe 13). Incubate the suspension on ice for 5–8 min. This step ruptures the plasma membrane of the protoplasts and releases intact vacuoles. Subsequently, centrifuge the lysate at 200× *g* for 5 min at 4 °C to pellet cellular debris and unbroken protoplasts. Carefully transfer the supernatant (enriched in vacuoles) to a fresh centrifuge tube. Finally, collect the supernatant and centrifuge it at 800× *g* for 10 min at 4 °C to pellet the vacuoles. Discard the supernatant and gently resuspend the pellet in vacuolar storage solution (Recipe 3).

*Notes:*

*1. Vacuoles can be preserved for at least 15 h under ice-cold conditions.*

*2. Under the microscope integrated with the NMT system, vacuoles are observed as empty, membrane-bound vesicles without visible internal structures, whereas protoplasts appear as intact, organelle-containing cells.*

**D. NH_4_^+^ influx assay**

**D1. Measurement of NH_4_^+^ influx at the root surface**

1. NH_4_^+^ calibration: Calibrate the non-invasive micro-test system (NMT Physiolyzer^®^) using test (Recipe 4a) and calibration solutions (Recipe 5a) at 25 °C. Consider a slope within the range of 58 ± 5 mV/decade as acceptable.

2. Sample preparation: Remove the sample to be measured from the culture medium. Gently press a single root of the seedling and fix it to the bottom of a Petri dish using filter paper strips and a sample-holding resin block. Then, add test solution to the Petri dish to completely immerse the root and allow the sample to equilibrate for 30 min under static conditions.

*Note: The volume of test solution added to the Petri dish varied according to the dish size: 4 mL for a 35 mm dish, 10 mL for a 60 mm dish, and 40 mL for a 90 mm dish. The equilibration time can be appropriately adjusted according to experimental requirements.*

**Figure 1.**
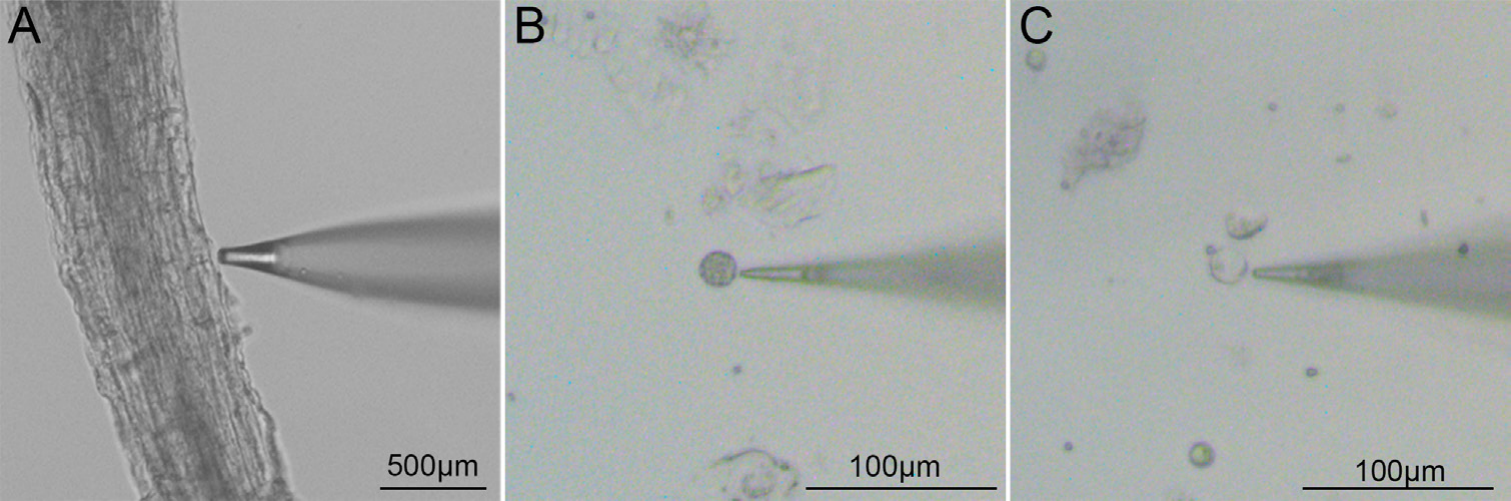
Microscopic images showing the probe and test sample. (A) Rice root. (B) Rice root protoplast. (C) Rice root vacuole.

3. NMT measurement: Transfer the Petri dish containing the sample to the microscope stage of the NMT instrument. Position an NH_4_^+^-selective microsensor (XY-STZ-NH4-C) equipped with the NMT Physiolyzer^®^ at the root elongation zone (1,000 μm from the root tip apex). Carefully position the sensor tip approximately 5 μm from the sample surface without making contact with the sample (Figure 1). Then, initiate data recording and continue for 5 min. Measure six biological replicates per experiment.

4. Data acquisition: Directly read and export the flow rate data using imFluxes V3.0 software. The unit of flow rate is pmol/cm^2^/s. The sign of the flow rate value (positive or negative) indicates the direction of NH_4_^+^ transport: a positive value represents NH_4_^+^ efflux from the root cell to the external solution, whereas a negative value represents the opposite direction.

**D2. Measurement of NH_4_^+^ influx in protoplast and vacuole samples**

**Figure 2.**
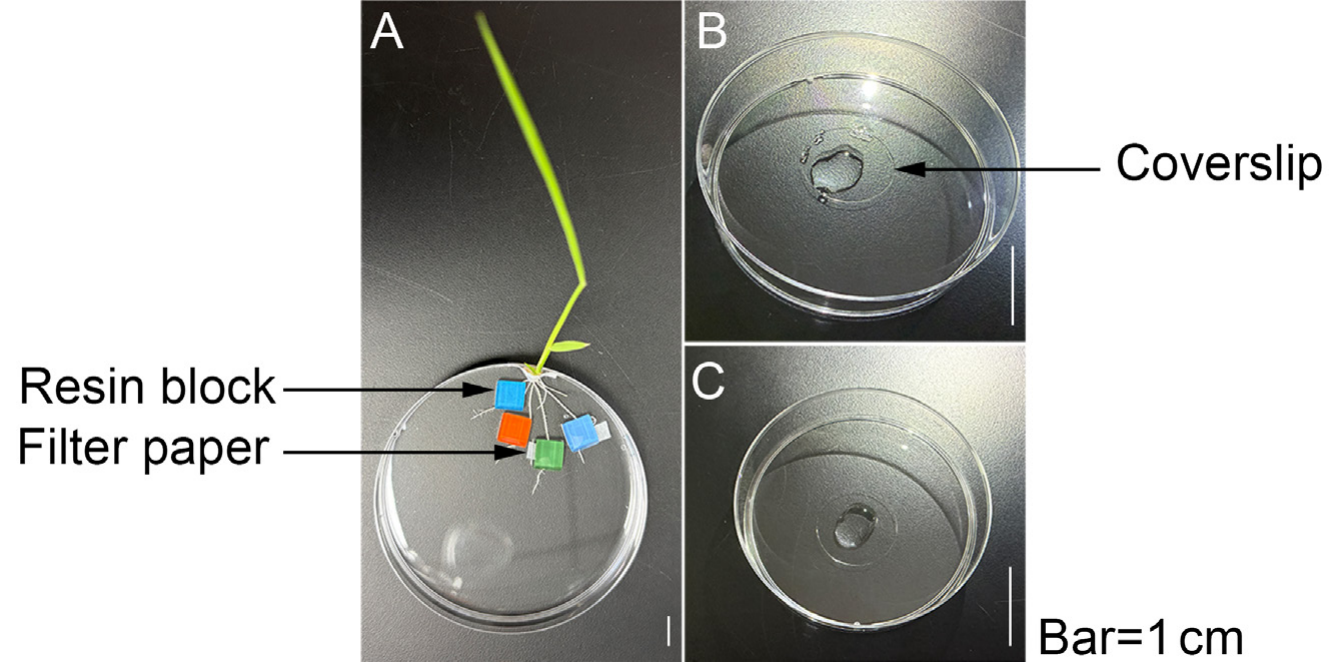
Schematic diagram of sample fixation. (A) Rice root. (B) Rice root protoplast. (C) Rice root vacuole.

1. NH_4_^+^ calibration: Calibrate the non-invasive micro-test system (NMT Physiolyzer**^®^**) with test (Recipe 4b for protoplasts and Recipe 4c for vacuoles) and calibration solutions (Recipe 5b for protoplasts and Recipe 5c for vacuoles) at 25 °C. Accept only a slope within 58 ± 5 mV/decade.

2. Sample preparation: Place a cell culture slide (Φ12 mm, BKMAM) at the bottom of a 36 mm Petri dish. Add a 100 μL drop of purified water between the slide and the dish bottom to help the slide stick to the dish. Drop 100 μL of protoplast/vacuoles suspension onto the center of the slide, then let the sample stand in a humid chamber for 10min to allow protoplasts/vacuoles to adhere to the slide surface. After that, add 5 mL of test solution to the Petri dish and let the sample equilibrate for 30min before measurement.

3. NMT measurement: Transfer the Petri dish with the sample to the microscope stage. Under the microscope, select individual protoplasts/vacuoles that attach to the dish bottom, appear morphologically intact and undamaged, and contain no visible internal contents. Position an NH^+^-selective microsensor (Xuyue) mounted on the NMT Physiolyzer^®^ approximately 5 µm above the target vacuole surface, making sure it does not touch the sample (Figure2). Start recording data for each protoplast/vacuole and continue for 5 min. Measure six replicates per experiment.

*Note: For protoplast and vacuole measurements, six biological replicates represent six individual protoplasts or vacuoles that meet the acceptance criteria under light microscopy, selected from the same isolation preparation derived from a single batch of rice seedling roots.*

4. Data acquisition: Read and export flow rate data directly with imFluxes V3.0 software. Express flow rates in pmol/cm^2^/s. The sign (positive or negative) indicates the transport direction of NH_4_^+^: a positive value means NH_4_^+^ efflux from the protoplast into the external solution (root apoplast or vacuole); a negative value means the opposite.

**E. NH_4_^+^ efflux assay**

**E1. Measurement of NH_4_^+^ efflux at the root surface**

1. NH_4_^+^ calibration: Calibrate the non-invasive micro-test system (NMT Physiolyzer^®^) with test (Recipe 6a) and calibration solutions (Recipe 7a) at 25 °C. Accept only a slope within 58 ± 5 mV/decade.

2. Sample pretreatment: Select rice seedlings of uniform growth at 12 days of age. One day before the measurement, transfer the seedlings to culture solutions either with or without high NH_4_^+^ stress and pretreat them for 24 h. Keep at least eight seedlings in each treatment group.

*Note: If NH_4_^+^ efflux inhibitors or other chemical reagents are required, add them at this step.*

3. Sample preparation: After pretreatment, remove the seedlings from the culture solution. Gently press a single root of each seedling against the bottom of a Petri dish and fix it in place using filter paper strips and a sample-holding resin block. Then, add test solution to the Petri dish until it completely immerses the root. Allow the sample to equilibrate under static conditions for 30 min.

4. NMT measurement: Transfer the Petri dish containing the sample to the microscope stage of the NMT instrument. Position an NH^+^-selective microsensor (Xuyue) mounted on the NMT Physiolyzer^®^ at the root elongation zone (approximately 1,000 μm from the root tip apex). Carefully place the sensor tip approximately 5 μm from the sample surface without touching the sample (Figure 1). Then, start data recording and continue for 5 min. Measure six biological replicates per experiment.

5. Data acquisition: Read and export the flow rate data directly using imFluxes V3.0 software. Express flow rates in pmol/cm^2^/s. The sign of the flow rate value (positive or negative) indicates the direction of NH_4_^+^ transport: a positive value means NH_4_^+^ efflux from the protoplast to the external solution (root apoplast); a negative value means the opposite direction.

**E2. Measurement of NH_4_^+^ efflux in protoplast and vacuole samples**

1. NH_4_^+^ calibration: Calibrate the non-invasive micro-test system (NMT Physiolyzer^®^) with test (Recipe 6b for protoplast and Recipe 6c for vacuole) and calibration solutions (Recipe 7b for protoplast and Recipe 7c for vacuole) at 25 °C. Accept only a slope within 58 ± 5 mV/decade.

2. Sample pretreatment and preparation

a. Control group: Mix a 50 μL aliquot of protoplast suspension with 50 μL of NH_4_^+^-free solution (Recipe 8). Let the mixture stand at room temperature for 1 h and 50 min. Then, transfer the mixture onto an adhesive slide placed at the bottom of a Petri dish and let it stand at room temperature for 10 min to allow protoplasts to adhere to the slide surface. Aspirate the liquid from the slide surface, then drop 100 μL of test solution onto the slide. After 50 s of treatment, immediately add 4.9 mL of test solution to the Petri dish (the final composition and concentration of the mixed solution should match those of the test solution).

b. High NH_4_^+^ transient treatment group: Mix a 50 μL aliquot of protoplast suspension with 50 μL of NH_4_^+^-free solution (Recipe 8). Let the mixture stand at room temperature for 1 h and 50 min. Then, transfer the mixture onto an adhesive slide placed at the bottom of a Petri dish and let it stand at room temperature for 10 min to allow protoplasts to adhere to the slide surface. Aspirate the liquid from the slide surface, then drop 100 μL of high NH_4_^+^ treatment solution (Recipe 9) onto the slide. After 50 s of treatment, immediately add 4.9 mL of NH_4_^+^-free solution to the Petri dish (the final composition and concentration of the mixed solution should match those of the test solution).

*Note: Perform the addition of 100 μL of treatment solution or test solution on the microscope stage and start the operation only after locating the target protoplast or vacuole.*

3. NMT measurement: Position an NH_4_^+^-selective microsensor (Xuyue) mounted on the NMT Physiolyzer^®^ approximately 5 μm above the surface of the target protoplast/vacuole, ensuring no contact with the sample (Figure 2). Start recording data for each protoplast/vacuole and continue for 5 min. Measure six replicates per experiment.

*Note: For protoplast and vacuole measurements, six biological replicates represent six individual protoplasts or vacuoles that meet the acceptance criteria under light microscopy, selected from the same isolation preparation derived from a single batch of rice seedling roots.*

4. Data acquisition: Read and export the flow rate data directly using imFluxes V3.0 software. Express flow rates in pmol/cm^2^/s. The sign of the flow rate value (positive or negative) indicates the direction of NH_4_^+^ transport: a positive value means NH_4_^+^ efflux from the protoplast to the external solution (root apoplast and vacuole); a negative value means the opposite direction.

## Data analysis

Net ion fluxes were measured using the NMT Physiolyzer^®^ system and directly exported using the imFluxes V3.0 software. Flux data are expressed in units of picomoles per square centimeter per second (pmol/cm^2^/s). The sign of the flux value indicates the direction of ion transport: for NH_4_^+^, a positive value denotes efflux from the protoplast or root cell into the extracellular space (root apoplast), while a negative value denotes influx in the opposite direction.

For each sample, fluxes were continuously recorded for 5 min, and the mean value was calculated for subsequent analysis. At least six biological replicates were used for each treatment group. All data are presented as means ± SD.

Statistical analyses were performed using GraphPad Prism 6. For pairwise comparisons between two experimental groups, Student’s t-test was employed. For multiple group comparisons, one-way or two-way analysis of variance (ANOVA) was performed, followed by Tukey’s post hoc test (α = 0.05).

No specific outlier exclusion criteria were applied; all measured values were included in the final dataset to preserve statistical power.

## Validation of protocol

This protocol has been validated and formally adopted in the following research articles:

- Di et al. [12]. Rapid induction of NH_4_^+^ efflux in rice roots under high-ammonium stress and its association with varietal differences in ammonium tolerance and ammonium-utilization efficiency. *New Phytologist.*
- Li et al. [7]. ISOPENTENYL TRANSFERASE3 activation triggers root ammonium hypersensitivity via the cytokinin-ARR10/ARR12-CAP1 signaling pathway, *Plant Physiology.*

## General notes and troubleshooting

**General notes**

1. The NH_4_^+^ and NO_3_- concentrations in the test solutions for measuring NH_4_^+^ influx at the root surface and in protoplasts should be consistent with those in the culture solution.

2. Significant differences exist in NH_4_^+^ flux data among different root types and root zones. Therefore, consistency in the root type and root zone selected for measurement should be ensured.

3. The distance between the sensor tip and the sample surface should be maintained at approximately 5 μm, and the tip must not contact the sample.

4. The test temperature should be kept stable, typically at 25 ± 0.5 °C. All solutions should be pre-equilibrated to this temperature before use.

5. To preserve statistical power, increase biological replicates rather than arbitrarily excluding outliers.

6. The threshold criterion for background noise consists of a dV fluctuation within ±1.5 µV (–1.5 µV < dV < +1.5 µV), with dV being the voltage differential between the two positions over one vibration cycle. This value directly reflects signal stability and the signal-to-noise ratio.

7. Identification and quality control of isolated protoplasts and vacuoles:

a. Morphological assessment under light microscopy: Light microscopic examination identifies acceptable protoplasts by the following morphological criteria: regular spherical shape, intact and smooth plasma membrane, dense cytoplasm with evenly distributed organelles (e.g., chloroplasts and nuclei), and full turgidity with no evidence of shrinkage, swelling, or rupture. For vacuoles, acceptance requires a round or nearly round shape, a thin and clearly outlined tonoplast, and a completely transparent lumen devoid of visible organelles or granular inclusions.

b. Vitality staining with neutral red: The vitality staining procedure mixes a 20 µL aliquot of the sample suspension with an equal volume of 0.1% (w/v) neutral red solution prepared in 0.3 M mannitol. After a 2–5 min incubation, microscopic observation reveals the staining patterns, which are interpreted according to the following criteria:

i. Viable protoplasts: The vacuolar compartment displays a characteristic cherry-red coloration, indicating the integrity of both the plasma membrane and the tonoplast, as well as the functional maintenance of an acidic luminal environment.

ii. Viable vacuoles: Uniform red staining throughout the entire vesicle appears, reflecting tonoplast integrity and the competence to sustain ionic gradients.

iii. Non-viable or damaged samples: Either no vacuolar staining (failure to accumulate the dye) or diffuse staining due to nonspecific dye adsorption to denatured cytoplasmic components or debris occurs.

A preliminary neutral red staining test on a small aliquot of each preparation prior to formal NMT measurements allows assessment of the overall batch quality. An intactness rate below 70% precludes continuation of the experiment and necessitates re-preparation of the sample.

c. Auxiliary validation using NMT: The present protocol uses the protoplast test solution (Recipe 4b) at pH 6.0 and the vacuole test solution (Recipe 4c) at pH 7.4. A substantial pH gradient naturally exists between the eukaryotic cytoplasm and the vacuolar lumen. Exposure of the sample to a mismatched test solution substantially alters this transmembrane gradient and thereby elicits aberrant NH_4_^+^ flux signals.

**Troubleshooting**

**Problem 1:** Large fluctuations in flow rate data.

Possible cause 1: Decreased sample activity, e.g., protoplasts or vacuoles stored for too long, or poor plant condition.

Solution 1: Measure protoplasts or vacuoles promptly after isolation and select healthy plants for measurement.

Possible cause 2: The microsensor is subject to external interference.

Solution 2: Ensure that the background signal fluctuations meet the acceptance criteria. If not, replace the microsensor with a new one.

**Problem 2:** Poor intra-group parallelism of data.

Possible cause 1: Large individual variation among samples, resulting in inconsistent physiological status.

Solution 1: Select experimental materials with similar physiological status or growth uniformity and increase the number of biological replicates.

Possible cause 2: Inconsistent root measurement sites across different samples.

Solution 2: Maintain the same root measurement site across different samples.

**Problem 3:** Low yield of intact root protoplasts.

Possible cause 1: Poor root vigor or mechanical damage caused by crushing during cutting.

Solution 1: Select seedlings with good growth status, such as 12-day-old rice seedlings. Use a sharp blade to cut the roots into 1 mm segments rather than mincing them, avoiding crushing.

Possible cause 2: Incomplete infiltration of the enzyme digestion solution.

Solution 2: Apply a vacuum to the enzyme digestion mixture in the dark for 30 min at the beginning of digestion or extend the digestion time.

Possible cause 3: Inappropriate centrifugation speed.

Solution 3: Reduce both the acceleration and deceleration rates of the centrifuge. It is recommended to set both acceleration and deceleration to level 3.

**Problem 4:** The NH_4_^+^-selective microelectrode exhibits excessive background noise.

Excessive background noise from the NH_4_^+^-selective microelectrode indicates unstable system signals and renders the measured ion-flux data unreliable. Troubleshoot in the following priority order:

Priority 1: Operational issues

a. Confirm that the reference electrode immersion depth in the solution exceeds 2 mm.

b. Confirm that the flow-rate microsensor immersion depth in the solution exceeds 2 mm.

c. Confirm complete immersion of the flow-rate microsensor holder in the filling solution.

d. Confirm complete immersion of the Ag/AgCl wire of the reference electrode in the 3 M KCl solution.

Priority 2: Component-related issues

a. Replace the microsensor with a new one.

b. Replace the sensor holder.

c. Replace the reference electrode.

Priority 3: Electrical issues

a. Check the voltage regulator for proper operation.

b. Check the grounding connections of the equipment (including the microscope illumination controller, preamplifier, and precision 3D positioning mechanical stage) for proper grounding.

c. Check for electromagnetic interference from other externally connected alternating current–powered devices.
